# Mechanical Strength and Hydration Characteristic of Multiple Common Waste-Blended Cement-Based Materials Cured by Electric-Induced Heating Curing Under Severely Cold Environments

**DOI:** 10.3390/ma18143220

**Published:** 2025-07-08

**Authors:** Lei Zhang, Ruisen Li, Sheng Li, Han Wang, Qiang Fu

**Affiliations:** 1 School of Water Conservancy and Civil Engineering, Northeast Agricultural University, Harbin 150030, Chinafuqiang@neau.edu.cn (Q.F.); 2 College of Civil Engineering & Architecture, Qingdao Agricultural University, Qingdao 266109, China; 3 School of Software Engineering, Jiangxi University of Science and Technology, Nanchang 330013, China; 4 School of Infrastructure Engineering, Nanchang University, Nanchang 330031, China

**Keywords:** electric-induced heating curing, winter concrete construction, waste materials, mechanical strength

## Abstract

To address the challenges of concrete construction in polar regions, this study investigates the feasibility of fabricating cement-based materials under severely low temperatures using electric-induced heating curing methods. Cement mortars incorporating fly ash (FA-CM), ground granulated blast furnace slag (GGBS-CM), and metakaolin (MK-CM) were cured at environmental temperatures of −20 °C, −40 °C, and −60 °C. The optimal carbon fiber (CF) contents were determined using the initial electric resistivity to ensure a consistent electric-induced heating curing process. The thermal profiles during curing were monitored, and mechanical strength development was systematically evaluated. Hydration characteristics were elucidated through thermogravimetric analysis (TG), X-ray diffraction (XRD), and Fourier-transform infrared spectroscopy (FTIR) to identify phase compositions and reaction products. Results demonstrate that electric-induced heating effectively mitigates the adverse effect caused by the ultra-low temperature constraints, with distinct differences in the strength performance and hydration kinetics among supplementary cementitious materials. MK-CM exhibited superior early strength development with strength increasing rates above 10% compared to the Ref. specimen, which was attributed to the accelerated pozzolanic reactions. Microstructural analyses further verified the macroscopic strength test results that showed that electric-induced heating curing can effectively promote the performance development even under severely cold environments with a higher hydration degree and refined micro-pore structure. This work proposes a viable strategy for polar construction applications.

## 1. Introduction

Concrete construction in polar region is hard to realize due to the following two aspects: (1) the severely low temperature hinders the hydration reaction inside the specimen, and (2) existing methods showed low efficiency while incurring large costs for the fabrication of concrete in negative-temperature environments [1,2,3]. Although many methods have been presented to solve the low-temperature concrete construction issue, they failed to address the dual challenges of material performance optimization and environmental sustainability in a holistic external condition [4,5,6]. Conventional approaches, such as external heating systems or chemical accelerators, often prioritize short-term strength development at early age and in a moderate environmental condition, with the temperature above −10 °C, and once the environment was lower than −10 °C the traditional curing methods exhibited very limited efficiency [7,8]. Moreover, the reliance on energy-intensive curing processes contradicts the global imperative for low-carbon construction, while the excessive use of chloride-based antifreeze additives raises concerns about steel corrosion and environmental contamination. These limitations underscore the urgent need to develop a novel curing method for the fabrication of cement-based materials in severely cold environments [9,10,11].

To address this issue, electric-induced heating has been introduced as an effective curing method to solve the construction problem of cement-based materials in severely cold environments [12,13,14]. More specifically, in 2018, Liu et al. conducted electric-induced heating curing experiments on cement mortar/graphene nanoplatelet (CM/GNP) composites and cement mortar/carbon nanofiber (CM/CNF) composites under −20 °C conditions with varying voltages. The results demonstrated that electric-induced heating curing effectively enhanced the mechanical properties of cementitious materials in subzero environments. Experimental data revealed that CM/GNP specimens subjected to 40 V electric-induced heating curing for 12 h achieved a compressive strength of 24.1 MPa, significantly exceeding the 3.5 MPa frost-resistant critical strength specified by ACI Committee 306R-10 [15,16]. Moreover, Tian et al. investigated the electrical properties of carbon fiber-reinforced conductive cement-based composites under negative temperatures, disclosing the adverse effects of electric-induced heating curing and freezing conditions on the specimen’s conductivity. The study demonstrated that CF could establish macroscopic conductive networks within specimens at subzero temperatures, substantially improving electrical performance [17]. Moreover, Tian et al. employed electric-induced heating curing to verify the long-term service performance requirements for cement-based materials in subzero environments. It was found that electric-induced heating curing enhanced both the mechanical properties and durability properties of the specimens, confirming reliable long-term strength development when early electric-induced heating cured specimens transitioned from −20 °C to room temperature conditions [18].

Moreover, it should be noted that in some rural regions the supply or the production of cement can be hard, while solid waste can be abundant. In this context, solid waste-derived supplementary cementitious materials (SCMs) present a promising paradigm shift for polar concrete technology. Industrial byproducts such as fly ash, slag, and metakaolin have demonstrated unique advantages in modifying cement hydration kinetics and microstructure evolutions under standard curing conditions [19,20,21]. Fly ash, a coal combustion residue, exhibits pozzolanic reactivity that can potentially mitigate thermal stress gradients through its delayed hydration characteristics [22,23]. Ground granulated blast slag (GGBS), with its latent hydraulic properties, may facilitate continued cementitious reactions under thermal constraints [24,25]. Metakaolin, a calcined clay product, stands out for its ability to refine pore structures and enhance interfacial transition zone integrity [26,27]. The use of these waste materials can not only relieve the environmental burden induced by the high CO_2_ emission of the production of cement but also improve the synergistical properties of cement-based materials. However, the specific reaction mechanical and performance development regularity are unknown for the specimens containing FA, GGBS, or MK under the action of electric-induced heating curing, especially in a severely cold environment.

To realize the fabrication of cement-based materials under severely low temperature towards concrete construction in polar region, this work explores the performance development of electric-induced heating cured cement mortar containing FA (FA-CM), GGBS (GGBS-CM), and MK (MK-CM) with environmental temperatures of −20 °C, −40 °C, and −60 °C. The curing temperatures of the specimens were recorded, and the mechanical strengths for the specimens were tested. To clarify the hydration characteristic of the specimens, Thermogravimetric (TG) analysis, Fourier-Transform Infrared Spectroscopy (FTIR) analysis, and BET analysis were conducted to elucidate the hydration products in the specimens cured by electric-induced heating curing under various negative temperatures.

## 2. Materials and Methods

### 2.1. Raw Materials

Ordinary Portland cement with the strength grade of 42.5 MPa was used, and FA, GGBS, and MK were employed as the substitution of cement. The chemical compositions of the main raw materials are illustrated in Table 1. The fine aggregate was fine silica sand with a size smaller than 1.18 mm. The Toray (Chuo City, Japan) produced carbon fibers (CFs) were used as the main conductive phase in CM to ensure the basic electrical conductivity of specimens with a length of 4 mm.

### 2.2. Mix Proportion and Preparation Process

In this work the cement was substituted by FA, GGBS, and MK at a constant mass ratio of 20%, and the individual waste materials were used as a singular cement substitution. The substitution rate was fixed at 20% to control the variables, so as to facilitate the horizontal comparison of the performance of different SCMs. The mix proportion of the specimen is listed in Table 2. To be specific, a constant water–binder ratio of 0.3 and sand–binder ratio of 1.5 were maintained throughout the formulation. The dry mixing of cementitious powders, fine aggregates, and CFs in a planetary mixer for 180 s was initially conducted to ensure the homogeneous dispersion. Subsequently, an aqueous solution containing polycarboxylic acid superplasticizer and water was incrementally introduced during continuous mixing, followed by an additional 3 min mixing to ensure the workability. Fresh mixtures were immediately cast into steel molds (40 mm × 40 mm × 160 mm) and consolidated through vibration compaction. Moreover, the fresh mixture was immediately placed in a negative temperature environment (−20 °C, −40 °C, and −60 °C) for further electric-induced curing. It is worth noting that the preparation process of all electric-induced heating cured specimens was carried out in a positive temperature environment (when the water is liquid), and the curing process was carried out in a negative temperature environment.

### 2.3. Curing Procedure

In extreme temperature environments the physical and chemical properties of the specimens will change rapidly during the curing process. This required the rapid strength formation of cement-based materials to ensure the quality of curing. As for the cement-based materials cured in this work, a two-day electric-induced heating curing process (a high-temperature curing technology) was conducted and compared with the conventional three-day standard curing method.

Room temperature (RT) curing was conducted as a comparison for the electric-induced heating curing. The curing temperature and humidity of RT curing were 20 ± 2 °C and >95%, respectively. The curing duration of RT curing was set to be 3 days.

The electric-induced heating curing was conducted for 2 days, with the environmental temperatures varying from −20 °C to −60 °C. The specimens were put into a refrigerator with a constant environmental temperature. The curing temperatures of electric-induced heating cured specimens were controlled within the range of 50–60 °C to ensure high-quality curing.

### 2.4. Test Methods

#### 2.4.1. Curing Temperature

**Figure 1:**
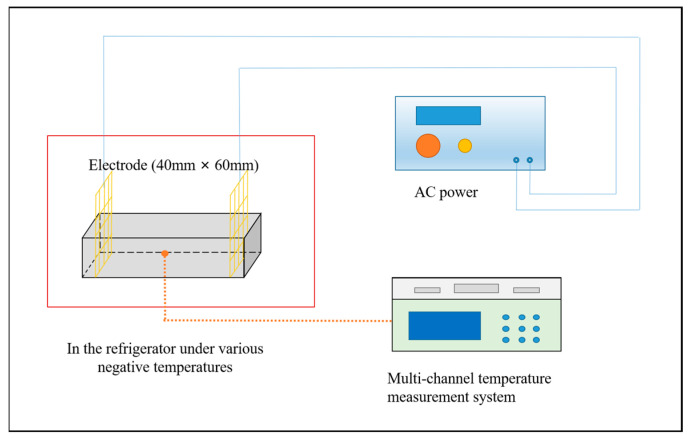
Temperature measurement illustration for electric-induced heating cured specimen.

Temperature is a pivotal curing parameter for electric-induced heating cured specimens, which can determine the curing quality for the cement-based materials. In this work, a thermocouple was inserted at the center of the CM specimen to record the curing temperature via a JK-16C, as depicted in Figure 1, Changzhou Jin’ailian Electronic Technology Co., Ltd., Changzhou, China.

#### 2.4.2. Mechanical Strength

Mechanical performance evaluation was evaluated based on GB/T 17671-2021 protocols, employing standardized testing configurations. Flexural properties were characterized using a servo-hydraulic testing machine (MTS Systems) under three-point bending configuration, maintaining loading rate at 50 N/s until structural failure. Compressive behavior analysis was conducted through uniaxial compressive loading at 2400 N/s strain rates. There were six parallel specimens for each condition, and the results of the mechanical strength were taken as the average of the six specimens.

#### 2.4.3. Electric Resistance

The electric resistance of the specimens was tested via a Tonghui TH2811D Digital Multimeter (Changzhou Tonghui Electronic Co., Ltd., Changzhou, China), and the test frequency was set to be 10 kHz, which was reported to effectively eliminate the interface resistance [28,29,30]. The electrical resistivity ρ was calculated in accordance with Equation (1), which is as follows:

(1) $$\rho =\frac{RS}{D}$$

where *S* is the effective contact area of the embedded electrodes inside the specimen, *D* is the effective distance between two electrodes, and *R* is the measured electric resistance of the CM specimen.

### 2.5. Microstructural Characterization

The hydration reaction of the specimen was terminated by immersing the broken specimens into ethanol. Then, the specimens were dried in a vacuum for 3 days at the temperature of 60 °C. The broken specimens were further crushed and screened for TG, FTIR, and BET analyses. A TG instrument (STA449F3, Netzsch Company, Selb, Germany) was employed for the TG test and the temperature rising rate was set to be 10 °C/min with a temperature range of 50 °C to 1000 °C, N_2_ was served as the protect atmosphere, and the sample mass was about 30 mg. FTIR was conducted to identify the phase inside the specimens, and the spectra were obtained within the range of 4000 cm^−1^ to 400 cm^−1^ with a resolution of 4 cm^−1^. Furthermore, Brunner–Emmett–Teller (BET) analysis was conducted to evaluate the micro-pore structure of the specimens, using an ASAP 2020, micromeritics company (Norcross, GA, USA).

## 3. Results and Discussion

### 3.1. CFs Contents Determination

**Figure 2:**
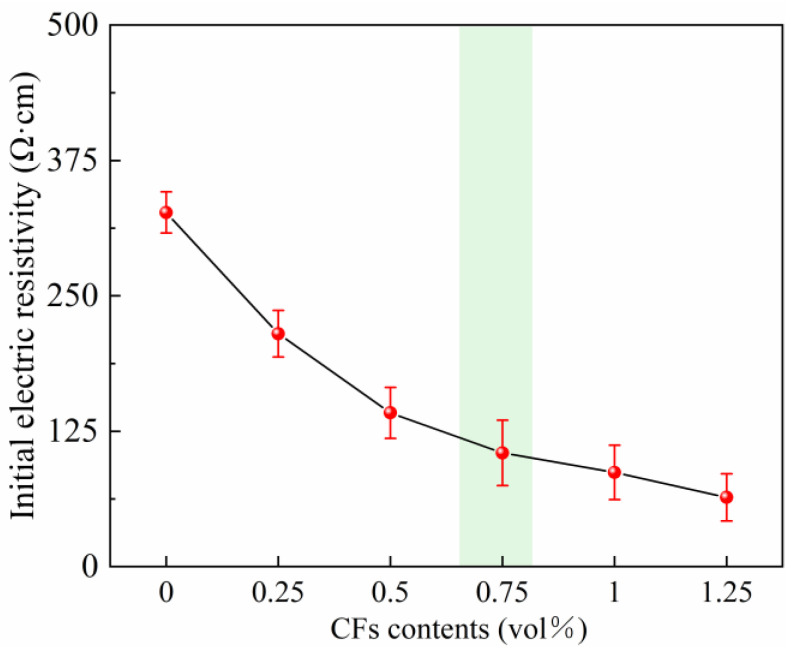
Initial electric resistivity development of CM specimen with various CFs contents.

The CF contents of the specimens were determined based on the initial electric resistivity measured by the two-electrode method. The CFs contents varied from 0 vol% to 1.25 vol%. Figure 2 illustrates the initial electric resistivity development regularity of CM specimens with various CF contents, and it can be found that the inclusion of CFs effectively improved the electric conductivity for the specimens. The detailed electric resistivity values of the specimens were 327 Ω·cm, 215 Ω·cm, 142 Ω·cm, 105 Ω·cm, 87 Ω·cm, and 64 Ω·cm, respectively, as the CF contents increased from 0 vol% to 1.25 vol%. The initial resistivity of the specimens within the range of 0–0.75 vol% CF content decreased rapidly, while the initial resistivity of the specimens within the range of 0.75–1.25 vol% CF content decreased slowly. Meanwhile, existing research indicated that the 0.75 vol% CF dosage showed a promising effect in forming a conductive network within the specimen [13], which was suitable for the performance development of cement-based materials cured by electric-induced heating curing. To be more specific, when the CF content was lower, the conducive network was not completely constructed, while a higher CF content may induce strength loss. Under this circumstance, we have decided on an optimal CF content of 0.75 vol% in this work to construct a fully connected network for the ongoing electric-induced heating curing process, which was also consistent with the published literature [31,32].

### 3.2. Curing Temperature Development

**Figure 3:**
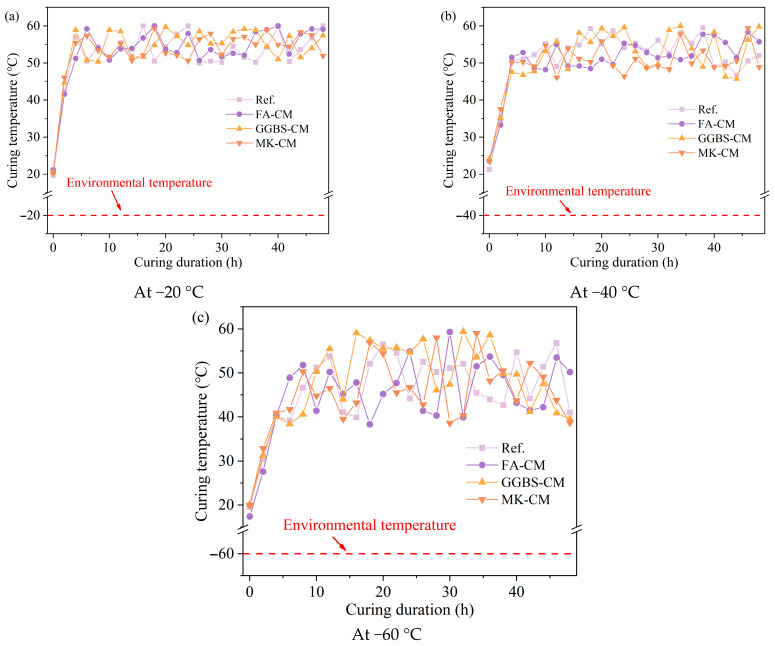
Curing temperature development of electric-induced heating cured specimens at various environmental temperatures: (**a**) at −20 °C, (**b**) at −40 °C, and (**c**) at −60 °C.

The curing temperature development of the specimens subjected to electric-induced heating curing is depicted in Figure 3. It can be found that the variation in the environmental temperature shows significant influence on the curing temperature development for the electric-induced heating cured specimens. To be specific, the curing temperatures were concentrated in the setting range of 50–60 °C when the environmental temperature was −20 °C, and the inclusion of waste materials showed no significant effect on the curing temperatures for the electric-induced heating cured specimens. The curing temperatures in the specimens cured under −40 °C were partially lower than those in the specimens cured under −20 °C, which was related to the fact that a higher energy supply was required to ensure the constant high-temperature curing condition. This was more obvious in the specimens cured by electric-induced heating curing under −60 °C as the curing temperatures fluctuated intensively at such a low environmental temperature within the range of 35–60 °C, as the electrical resistivity can be significantly influenced under such a big temperature difference between the electric heating cured specimen and the environment. Also, the required energy consumption is huge to ensure a constant temperature development for the specimen, which indicates that a modified electric-induced heating curing regime is required to realize the fabrication of cement-based materials in such a harsh environment.

### 3.3. Compressive and Flexural Strengths

**Figure 4:**
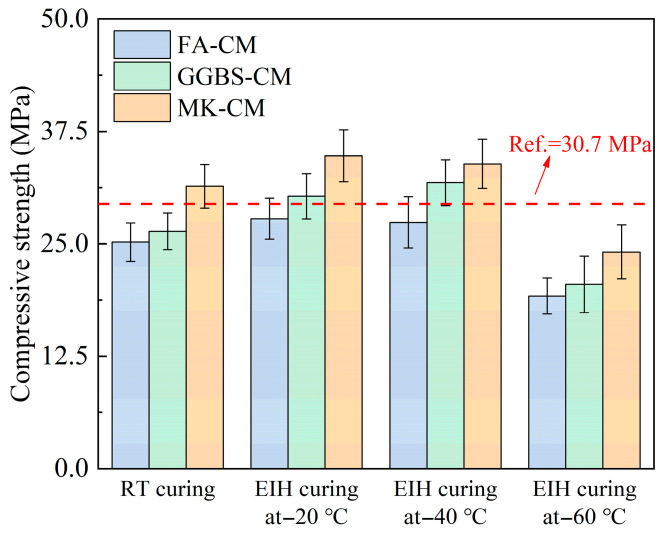
Compressive strengths of electric-induced heating cured CM specimens under various negative temperatures.

The compressive strengths of the electric-induced heating cured specimens under different environmental temperatures are depicted in Figure 4. As depicted in Figure 4, the Ref. specimen (pure CM cured by 3 days of RT curing) showed a compressive strength of 30.7 MPa, which became 25.2 MPa, 26.4 MPa, and 31.4 MPa for the specimens containing 20% FA, 20% GGBS, and 20% MK, respectively, under the same curing conditions. It can be found that the inclusion of FA and GGBS was detrimental to the strength of RT-cured specimens, which was related to the low hydration reaction activity of these two cementitious materials at an early age. However, MK can effectively improve the early-age strength of CM under RT curing conditions due to its high activity. Moreover, the implementation of electric-induced heating curing can partly compensate for the adverse effect induced by the inclusion of water materials, even in a negative temperature environment. To be specific, the compressive strengths of FA-CM, GGBS-CM, and MK-CM cured by electric-induced heating curing were 27.8 MPa, 30.3 MPa, and 34.8 MPa under −20 °C. These values became 27.4 MPa, 31.8 MPa, and 33.9 MPa with the environmental temperature of −40 °C, which were further reduced to 19.2 MPa, 20.5 MPa, and 24.1 MPa under −60 °C. It can be concluded that the electric-induced heating curing effectively enhanced the compressive strengths of CM specimens by creating a high-temperature within the negative temperature environment. And the compressive strengths of the GGBS-CM and MK-CM specimens were higher than the Ref. specimens, especially for the MK-CM specimens where the compressive strengths were improved by 13.4% and 10.4%, respectively, at the environmental temperatures of −20 °C and −40 °C, highlighting the feasibility of electric-induced heating curing for preparing cement-based materials even under in a severely cold environment.

However, it should be noted that the strength development of electric-induced heating cured specimens was hindered under −60 °C, which can be related to the fact that large temperature fluctuations significantly affected the performance development of cement-based materials in harsh environments. Based on this strength development law, the electric-induced heating curing regime should be further modified to adapt to the more severe environment in polar region or even in space.

**Figure 5:**
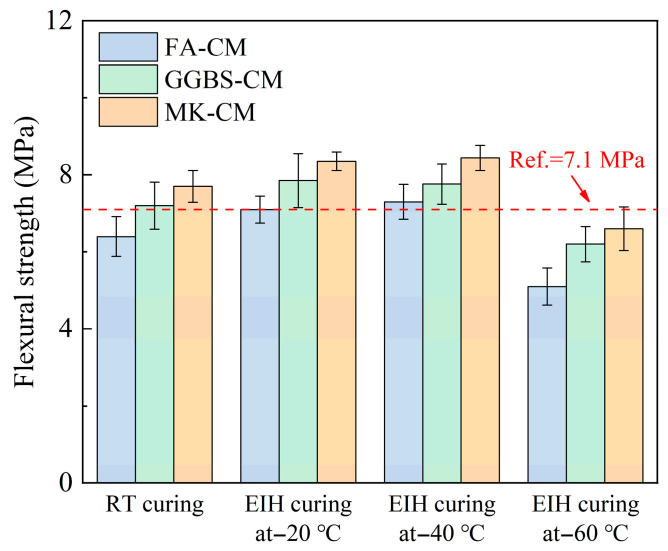
Flexural strengths of electric-induced heating cured CM specimens under various negative temperatures.

Moreover, the flexural strength depicted a similar development regularity with the compressive strength, as shown in Figure 5, showing that electric-induced heating curing can ensure a good flexural strength development trend, especially for the GGBS-CM and MK-CM specimens cured under −20 °C and −40 °C. The flexural strengths of RT cured specimens were 7.1 MPa, 6.4 MPa, 7.2 MPa, and 7.7 MPa, respectively, for the Ref., FA-CM, GGBS-CM, and MK-CM specimens. These values became 7.1 MPa, 7.9 MPa, and 8.4 MPa for the electric-induced heating cured FA-CM, GGBS-CM, and MK-CM specimens under −20 °C. At environmental temperatures of −40 °C and −60 °C, the flexural strengths of the electric-induced heating cured FA-CM, GGBS-CM, and MK-CM specimens were 7.3 MPa, 7.8 MPa, and 8.4 MPa (−40 °C) and 5.1 MPa, 6.2 MPa, and 6.6 MPa (−60 °C), respectively. The flexural strength development regularity further proved the feasibility of electric-induced heating curing on the fabrication of cement-based materials.

### 3.4. TG Analysis

**Figure 6:**
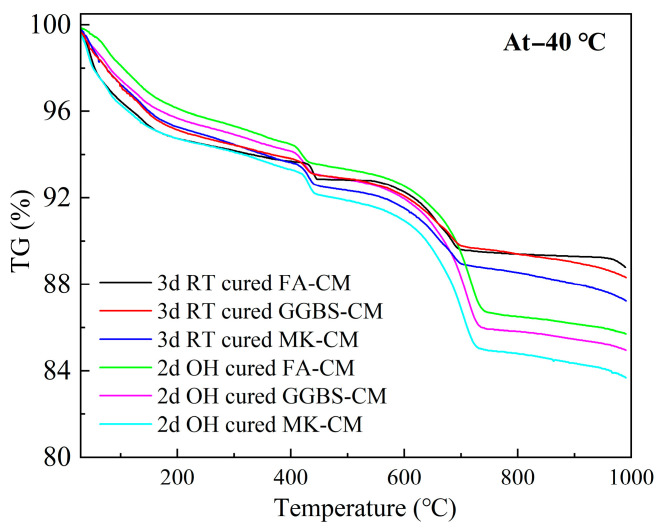
TG analysis of CM specimens subjected to various curing conditions.

TG analysis is an effective method to evaluate the hydration degree of cement-based materials by quantitively calculating the phase-mass inside the specimens. The TG curves of the specimens treated by different curing conditions are shown in Figure 6. Three main mass loss peaks can be found in the TG curves, in general, where the thermal decomposition within the range of 80–200 °C is related to the decomposition of the water and the dehydration of C-S-H and AFt [33,34]. The peak shown in 380–450 °C is the decomposition of Ca(OH)_2_ [35,36]. Furthermore, the mass loss exhibited in 650–800 °C is related to the decomposition of CaCO_3_ [37,38].

To better understand the hydration reaction inside the specimens, the TG analysis was conducted among the electric-induced heating cured specimens with the environmental temperature of −40 °C, and the RT cured specimens were used as the reference. It can be found that the mass losses after 2 days in the electric-induced heating cured specimens were always higher than those in the specimens cured by 3 days of RT curing, indicating the more intense hydration reaction in the electric-induced heating cured specimens. This can explain the high mechanical strengths in the specimens. Moreover, it should be noted that the mass loss in the specimens followed the order of MK-CM > GGBS-CM > FA-CM, and this was related to the low hydration reaction of FA, especially at an early age [39,40].

### 3.5. FTIR Analysis

**Figure 7:**
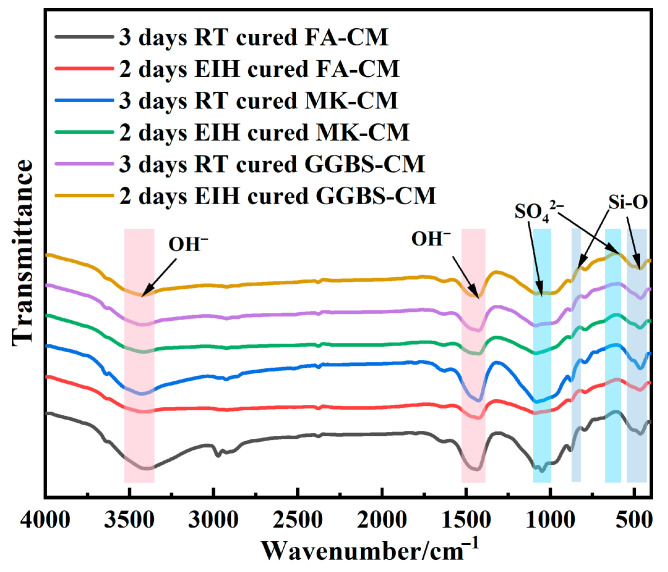
FTIR analysis of specimens treated by different curing conditions.

The FTIR experiment in the experiment was used as a qualitative method rather than a quantitative one, as we only needed to determine whether certain products exist in the specimen through the peaks rather than to determine the quantity of the products by observing the position of the peak height. Based on the infrared spectrum analysis results of the concrete specimens composed of three common waste materials, FA, GGBS, and MK, after standard curing and electric-induced heating curing (as shown in Figure 7), the vibration peak characteristics were summarized. The peaks around 500 cm^−1^ and 900 cm^−1^ were caused by the bending and tensile vibrations of the Si-O bond in the silicate. The weak peaks near 650 cm^−1^ and 1000 cm^−1^ were caused by the stretching and bending vibrations of sulfate ions. The peaks around 1600 cm^−1^ and 3500 cm^−1^ were caused by the tensile and bending vibrations inside the crystal water. Through the analysis of the data and analysis results in the figure, it can be seen that the internal chemical bond reactions of the three materials corresponding to different curing methods show consistent characteristics, which confirms that the final products of the concrete specimens were consistent in composition and structure. This indicates the safety and consistency of electric-induced heating for preparing CMs at severely low temperatures.

### 3.6. Pore Structure Analysis

**Figure 8:**
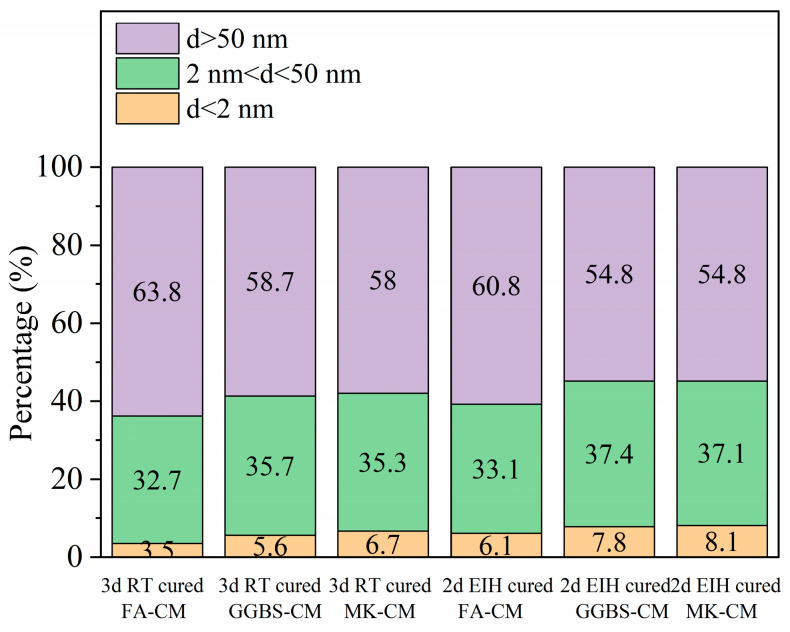
Accumulated pore volume of electric-induced heating and RT cured specimens.

The BET pore structure analysis was further conducted to disclose the micro-pore structure in electric-induced heating cured specimens. Figure 8 exhibits the accumulated pore volume of the specimens cured by RT curing and electric-induced heating curing at the environmental temperature of −40 °C. It can be observed that electric-induced heating curing can effectively refine the micro-pore structure of the specimens as the percentage of pores with a diameter < 2 nm was always higher in electric heating cured specimens, and the percentage of pores with a diameter > 50 nm was always lower. This indicates the beneficial effect of electric-induced heating curing on improving the pore structure in a high-temperature curing condition, which was in consistent with the mechanical strength and TG hydration analysis results.

## 4. Conclusions

In this work, the feasibility of electric-induced heating curing as an innovative strategy to overcome ultra-low temperature challenges in polar concrete construction was detailed, with FA, GGBS, and MK used as the cement substitution to lower the carbon emission. By incorporating carbon fiber (CF) to regulate resistivity and applying controlled electric heating, FA-CM, GGBS-CM, and MK-CM achieved effective hydration and strength development even at temperatures as low as −60 °C. Notably, MK-CM exhibited superior early strength enhancement (MK-CM achieved strength increments of 13.4% and 10.4% versus the RT-cured reference at −20 °C and −40 °C, respectively), which has been attributed to accelerated pozzolanic reactions and refined pore structures, as validated by TG and BET analyses. The distinct performance of supplementary cementitious materials underscores the importance of tailored material selection for extreme environments. These findings highlight the potential of electric-induced heating curing to enable high-performance cementitious materials in polar regions, offering a practical solution for infrastructure development under severe climatic constraints. Future work should explore the long-term durability and scalability to advance sustainable polar construction technologies.

## Figures and Tables

**Table 1:** Chemical compositions of the raw materials (wt%).

	SiO_2_	Al_2_O_3_	Fe_2_O_3_	CaO	MgO
Cement	20.58	5.03	3.38	63.32	2.01
MK	54	44	<0.5	<0.1	<0.05
FA	48.41	17.88	3.96	8.45	0.89
GGBFS	35.23	15.38	13.12	27.41	7.79

**Table 2:** Mixing proportions of the specimens.

Water (g)	Cement (g)	Common Waste (g)	Silica Sand (g)	CFs Contents
60	160	40	300	0 vol%
60	160	40	300	0.25 vol%
60	160	40	300	0.5 vol%
60	160	40	300	0.75 vol%
60	160	40	300	1.0 vol%
60	160	40	300	1.25 vol%

## Data Availability

The original contributions presented in this study are included in the article. Further inquiries can be directed to the corresponding author.
